# Cytoskeletal Signaling: Is Memory Encoded in Microtubule Lattices by CaMKII Phosphorylation?

**DOI:** 10.1371/journal.pcbi.1002421

**Published:** 2012-03-08

**Authors:** Travis J. A. Craddock, Jack A. Tuszynski, Stuart Hameroff

**Affiliations:** 1 Department of Physics, University of Alberta, Edmonton, Canada; 2 Department of Experimental Oncology, Cross Cancer Institute, Edmonton, Canada; 3 Departments of Anesthesiology and Psychology, Center for Consciousness Studies, University of Arizona, Tucson, Arizona, United States of America; University of Salzburg, Austria

## Abstract

Memory is attributed to strengthened synaptic connections among particular brain neurons, yet synaptic membrane components are transient, whereas memories can endure. This suggests synaptic information is encoded and ‘hard-wired’ elsewhere, e.g. at molecular levels within the post-synaptic neuron. In long-term potentiation (LTP), a cellular and molecular model for memory, post-synaptic calcium ion (Ca^2+^) flux activates the hexagonal Ca^2+^-calmodulin dependent kinase II (CaMKII), a dodacameric holoenzyme containing 2 hexagonal sets of 6 kinase domains. Each kinase domain can either phosphorylate substrate proteins, or not (i.e. encoding one bit). Thus each set of extended CaMKII kinases can potentially encode synaptic Ca^2+^ information via phosphorylation as ordered arrays of binary ‘bits’. Candidate sites for CaMKII phosphorylation-encoded molecular memory include microtubules (MTs), cylindrical organelles whose surfaces represent a regular lattice with a pattern of hexagonal polymers of the protein tubulin. Using molecular mechanics modeling and electrostatic profiling, we find that spatial dimensions and geometry of the extended CaMKII kinase domains precisely match those of MT hexagonal lattices. This suggests sets of six CaMKII kinase domains phosphorylate hexagonal MT lattice neighborhoods collectively, e.g. conveying synaptic information as ordered arrays of six “bits”, and thus “bytes”, with 64 to 5,281 possible bit states per CaMKII-MT byte. Signaling and encoding in MTs and other cytoskeletal structures offer rapid, robust solid-state information processing which may reflect a general code for MT-based memory and information processing within neurons and other eukaryotic cells.

## Author Summary

Memory is understood as strengthened synaptic connections among neurons. Paradoxically components of synaptic membranes are relatively short-lived and frequently re-cycled while memories can last a lifetime. This suggests synaptic information is encoded at a deeper, finer-grained scale of molecular information within post-synaptic neurons. Long-term memory requires genetic expression, protein synthesis, and delivery of new synaptic components. How are these changes guided on the molecular level? The calcium-calmodulin dependent protein kinase II (CaMKII) has been heavily implicated in the strengthening of active neural connections. CaMKII interacts with various substrates including microtubules (MTs). MTs maintain cellular structure, and facilitate cellular cargo transport, effectively controlling neural architecture. Memory formation requires reorientation of this network. Could CaMKII-MT interactions be the molecular level encoding required to orchestrate neural plasticity? Using molecular modeling and electrostatic profiling, we show a precise matching between the spatial dimensions, geometry and electrostatics of CaMKII and MTs, and calculate the potential information capacity and bio-energetic parameters of such interactions. Results suggest signaling and encoding in MTs offers rapid, robust information processing with a large potential for memory storage, reflecting a general code for MT-based memory in neurons and other eukaryotic cells.

## Introduction

### LTP, CaMKII and Microtubules

The brain's ability to learn and store memory is understood in terms of changes in synaptic connections between neurons: ‘synaptic plasticity’ [1]. This is supported by the paradigm of ‘long-term potentiation’ (LTP) in which repetitive pre-synaptic stimulation increases post-synaptic sensitivity and strengthens synapses (e.g. the adage “neurons that fire together, wire together”). LTP is supported experimentally *in vitro* [2], [3], and may occur over many brain regions [4] as a common feature of excitatory synapses [5].

Below the level of the synapse, LTP involves complex gene expression, protein synthesis and recruitment of new receptors or even synapses [6]. Generally, however, synaptic plasticity is viewed in terms of changes in function, location and/or number of post-synaptic receptors and ion channels. However, synaptic receptors and channel proteins are transient, being synthesized and degraded in the protein lifecycle, and yet memories can last lifetimes. Therefore, information pertinent to memory must be stored elsewhere, yet remain able to regulate synaptic plasticity.

LTP involves the neurotransmitter glutamate binding to post-synaptic receptors, opening calcium channels to allow influx of calcium ions (Ca^2+^) to dendritic spines [7], shafts and neuronal cell body. Within dendritic spines, inflow of Ca^2+^ results in activation of multiple enzymes including protein kinase A [8], protein kinase C [9] and Ca^2+^-calmodulin kinase II (CaMKII) [10]. These enzymes, in turn, interact with (e.g. by phosphorylating) various intra-neuronal molecules, presumably for storage and processing of synaptic information. These CaMKII-phosphorylated protein structures must then, in some as-yet-unknown way, encode memory and regulate synaptic plasticity.

CaMKII has 12 kinase domains (6 on one side, 6 on the other), normally folded tightly in an inactive state to an association domain. Ca^2+^ influx to post-synaptic neurons binds calmodulin (CaM) to form Ca^2+^/CaM complexes, versatile regulators of multiple proteins and enzymes. Ca^2+^/CaM complexes activate CaMKII kinase regions allowing them to extend from the association domain and enable phosphorylation of a substrate protein, i.e. transfer of a phosphate group (through ATP hydrolysis) to a serine or threonine residue on a protein.

As Ca^2+^ influx can activate any, or all, of the 12 kinases, CaMKII has been suggested to record synaptic activity, retaining a ‘memory’ of past Ca^2+^ influx events in terms of activated phosphorylation states [11]. CaMKII may also auto-phosphorylate, in which one kinase phosphorylates an adjacent kinase on a single holoenzyme, resulting in prolonged CaMKII activation and possible information processing after initial Ca^2+^ influx. CaMKII is effectively a Ca^2+^ triggered synaptic memory device [12]. How and where information is encoded by CaMKII to be stored, and utilized to regulate synaptic plasticity remain open questions.

Following synaptic activity, CaMKII is rapidly distributed throughout post-synaptic neuronal dendrites and cell bodies [13]. The active sites of substrate phosphorylation on CaMKII kinase regions are referred to as ‘S’ sites and ‘T’ sites, apparently related to shorter and longer-term phosphorylation, respectively [14]. Single point mutation of amino acid threonine 286 in CaMKII results in impaired Ca^2+^ dependent alterations in synaptic plasticity, as well as in learning and memory in mouse models [10]. CaMKII phosphorylation of post-synaptic protein substrates is a likely mechanism for memory encoding/storage and regulation of synaptic plasticity.

Synaptic plasticity involves neuronal differentiation, movement, synaptogenesis and up- and down-regulation, all requiring, in one way or another, MTs, major structural components of the cytoskeleton. MTs are composed of tubulin, 110 kD protein hetero-dimers which self-assemble into hollow cylinders 25 nm in outer and 15 nm in inner diameter. MT walls have been crystallographically characterized as forming two types of hexagonal lattices (A-lattice and B-lattice) with helical winding patterns, including those following Fibonacci geometry [15]. Each tubulin dimer in MT lattices may occupy different states, and interact with neighbor tubulin dimer states, suggesting to a number of authors [16]–[20] that MTs process information in terms of tubulin states, and function as computational devices, e.g. molecular automata (‘microtubule automata’).

MTs in neuronal axons are arranged in continuous, uninterrupted parallel bundles. However, MTs in dendrites and neuronal cell bodies are uniquely arranged in mixed polarity, anti-parallel arrays of interrupted MTs, interconnected by MT-associated proteins (“MAPs”) including MAP2, whose activities are implicated in learning ([21] and references therein).

Various types of MAPs include those which cross-bridge MTs into scaffold-like networks defining neuronal architecture, and motor proteins (e.g. dynein, kinesin) which transport molecular cargo along MTs for delivery to specific synaptic locations. In most cells, MTs are labile and tend to undergo cycles of assembly and disassembly. However in neuronal dendrites and cell bodies, MTs are capped and stabilized by specialized MAPs including MAP-2 [22], [23].

MTs in brain neurons may also be stabilized by post-translational modifications. α-tubulin isotypes have either tyrosine, or glutamate at their C-terminal ends. Generally, MTs with abundant glutamate-terminating tubulins are stable, while MTs with many tyrosine-terminating tubulins are labile and short-lived [24], [25]. Terminal tyrosine may be removed (“detyrosination”) via tubulin carboxypeptidase, yielding glutamate-terminating tubulin and giving rise to disassembly-resistant MTs [26]. Stable MTs composed of glutamate-terminating α-tubulin appear to interact via MAPs with another, extremely stable cytoskeletal component, namely intermediate filaments [27]. Thus short-term memory stored in MTs may be further encoded and hard-wired in neurofilaments for long-term memory.

Evidence for MT-based information processing includes correlations between tubulin production and visual processing [28], MT signaling between membrane proteins and cell nucleus [29], [30], and MT-MAP alterations correlating with memory, novel learning and cytoskeletal remodeling [31]. Deficits in cytoskeletal function affect learning and memory [20], with the best-known correlation being disruption of MTs and resulting neurofibrillary tangles in brains of Alzheimer's disease patients.

In this study we performed molecular mechanics modeling and electrostatic profiling of binding and phosphorylation between activated CaMKII holoenzyme kinase domains and tubulin lattices in MTs, and analyzed their information processing capabilities.

## Results

### Calcium Calmodulin Kinase II

**Figure 1:**
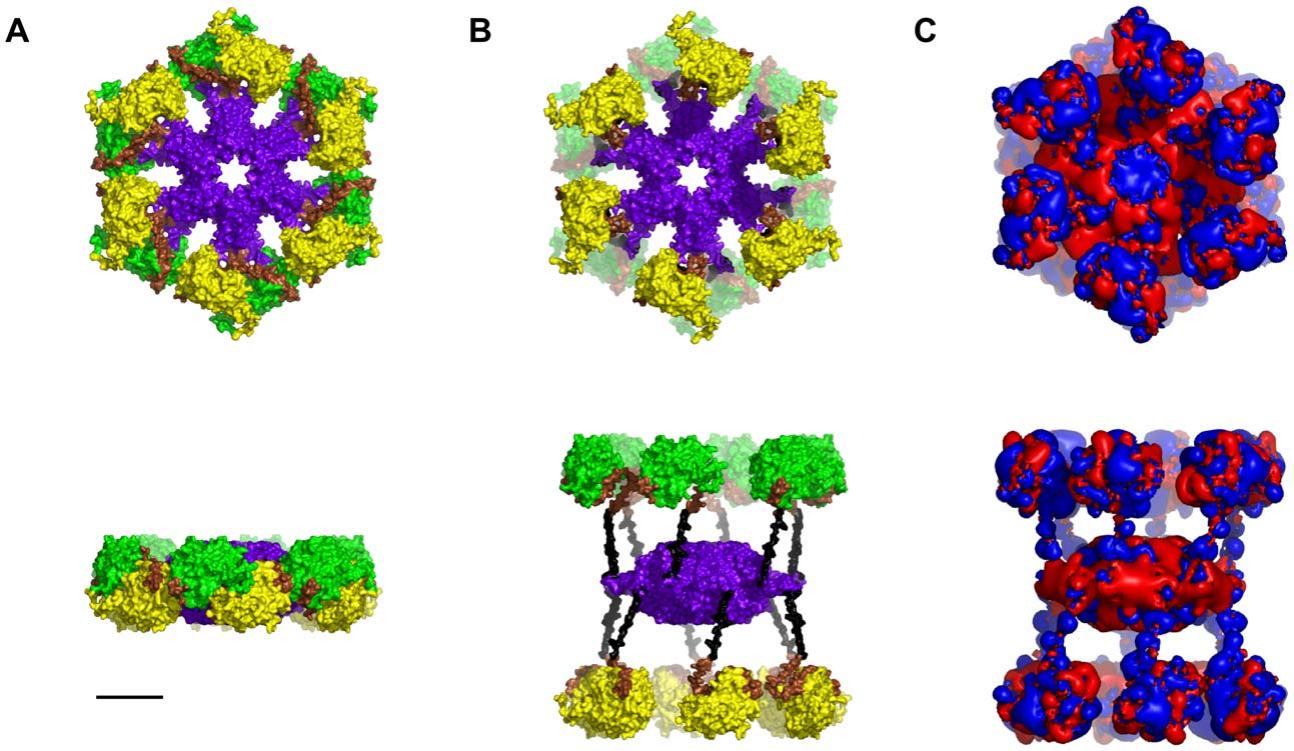
The CaMKII holoenzyme. Top - Face View, Bottom - Side View. (A) Not activated. Yellow/Green - Kinase domains, Purple - Association domain, Brown –Autoregulatory domain, Black – Linker domain. (B) Activated, (C) Electrostatic map: Blue +0.5 kT/e, Red −0.5 kT/e. Scale bar 5 nm.

CaMKII is a holoenzyme with twelve kinase regions in two hexagons sandwiched around a central association domain (see Figure 1 A). Activated by Ca^2+^/CaM, the kinases extend outward from the association domain, remaining tethered by linker regions (see Figure 1 B).

The phosphorylation process takes place on the inner surface of the kinase domains, in ‘hydrophobic groove’ regions characterized by valine 208 and tryptophan 237 (and, in the inactive state, occupied by threonine 286 on the autoregulatory region). The S-T hydrophobic phosphorylation site is exemplified by valine 208. Symmetric arrangement of the kinase regions provides a unique electrostatic profile in which each kinase region presents a positive potential region surrounded by negatively charged surfaces (see Figure 1 C). Substrates with reciprocal patterns of negatively charged regions on a positive background are excellent candidates for attractive electrostatic interactions.

### Tubulin and Microtubule Lattice

**Figure 2:**
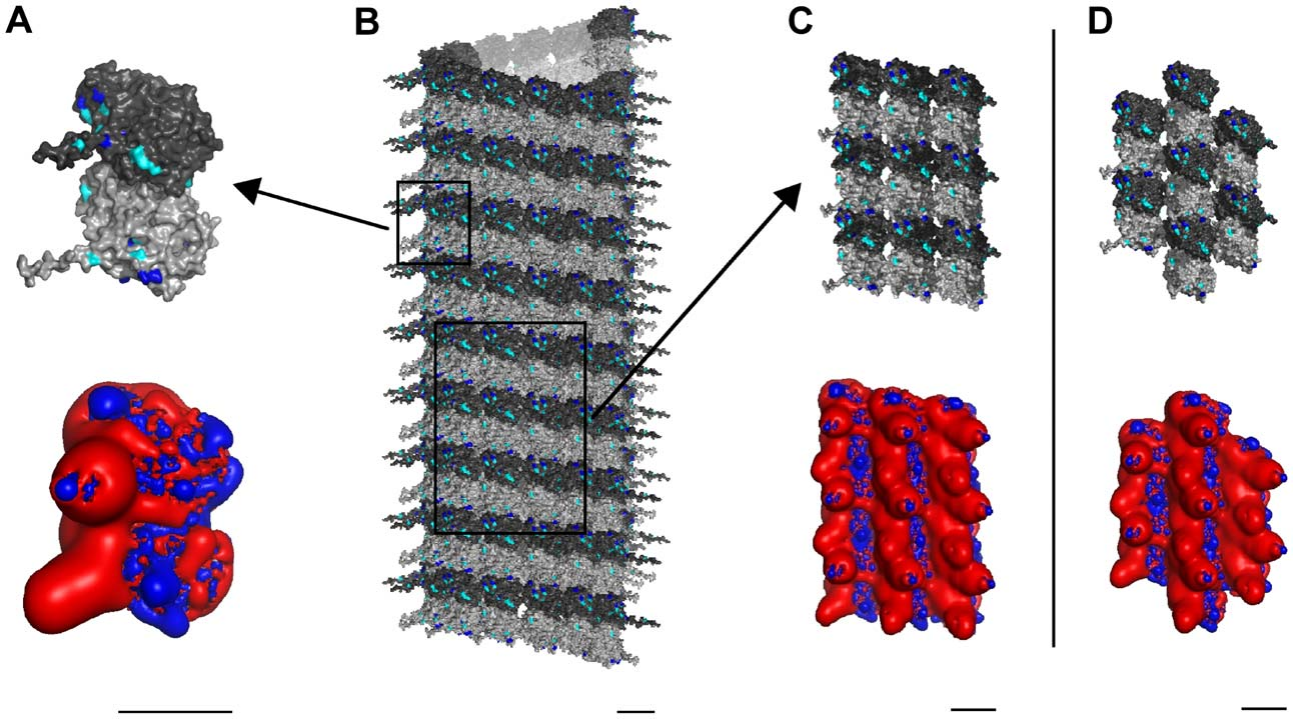
Three scales of MT constituent protein tubulin with electrostatic maps. Light gray – α-tubulin, Dark gray – β-tubulin. (A) Top – Single tubulin dimer with potential phosphorylation sites highlighted: Blue - Threonine, Cyan – Serine. Bottom – Electrostatic map: Blue +0.5 kT/e, Red −0.5 kT/e. (B) Cylindrical B-lattice MT. (C) Top − 9 dimer B-lattice patch. Bottom – Electrostatic map. (D) Top – 7 dimer A-lattice patch. Bottom – Electrostatic map. Scale bars 5 nm.

As previously described, MTs are cylindrical polymers of hetero-dimer tubulin proteins, each composed of α- and β-tubulin monomers (see Figure 2 A). Tubulin dimers self-assemble into MTs, which are hollow cylinders of 13 linear tubulin chains known as ‘protofilaments’ (see Figure 2B). Side-to-side electrostatic interactions between tubulins in parallel protofilaments comprising the cylinder result in two types of skewed hexagonal lattices and helical winding pathways, the so-called A-lattice and B-lattice [32] (see Figure 2 C & D).

Overall, tubulin has a large net negative surface charge with almost half of the negatively charged residues located on the C-terminal tail of each monomer, which extend from the surface into the cytoplasm (see Figure 2A). This net electrostatic charge is, obviously, neutralized by counter-ions in the cytoplasm. Surface charges differ between α- and β monomers resulting in a net dipole moment pointing from the α- towards the β-monomer. Thus, MTs have net dipole moments, reflecting alignment of tubulin dipoles, and a unique skewed hexagonal electrostatic pattern of highly negatively charged regions surrounded by a positive background, dependent on the MT lattice type (see Figure 2 C & D).

### CaMKII Phosphorylation of Tubulin

Generally, CaMKII shows a preference for phosphorylating protein substrates at the amino acid sequence arginine-X-X-serine/threonine, where X can be any amino acid (although exceptions occur [33]). Wandosell et al. [34] showed that free, unpolymerized α- and β-tubulin are phosphorylated by activated CaMKII on or near the C-terminal region (beyond residue 306), resulting in tubulin conformational change, inhibition of assembly and inability to bind MT-associated protein 2 (MAP2). Multiple serine and threonine residues capable of being phosphorylated exist in this region (see Figure 2) however, the exact CaMKII phosphorylation site, or sites, on tubulin are unknown. Several sites on α- and β-tubulin follow this consensus sequence, however only one falls within the C-terminal region [35]. That site involves threonine 312 located not on the surface, but buried in the cleft between the α- and β-monomers. Another potential site is serine 444 on the βIII-tubulin C-terminal tail. Experiments suggest that phosphorylation of mammalian β-tubulin is restricted to the βIII isotype [27], which is found predominately in neurons. While alternate sites on βIII-tubulin have been argued for [36], it is clear that phosphorylation of tubulin by CaMKII holoenzymes occurs, though the precise sites remain unclear.

### Quaternary Protein Structure Hexagonal Geometry

**Figure 3:**
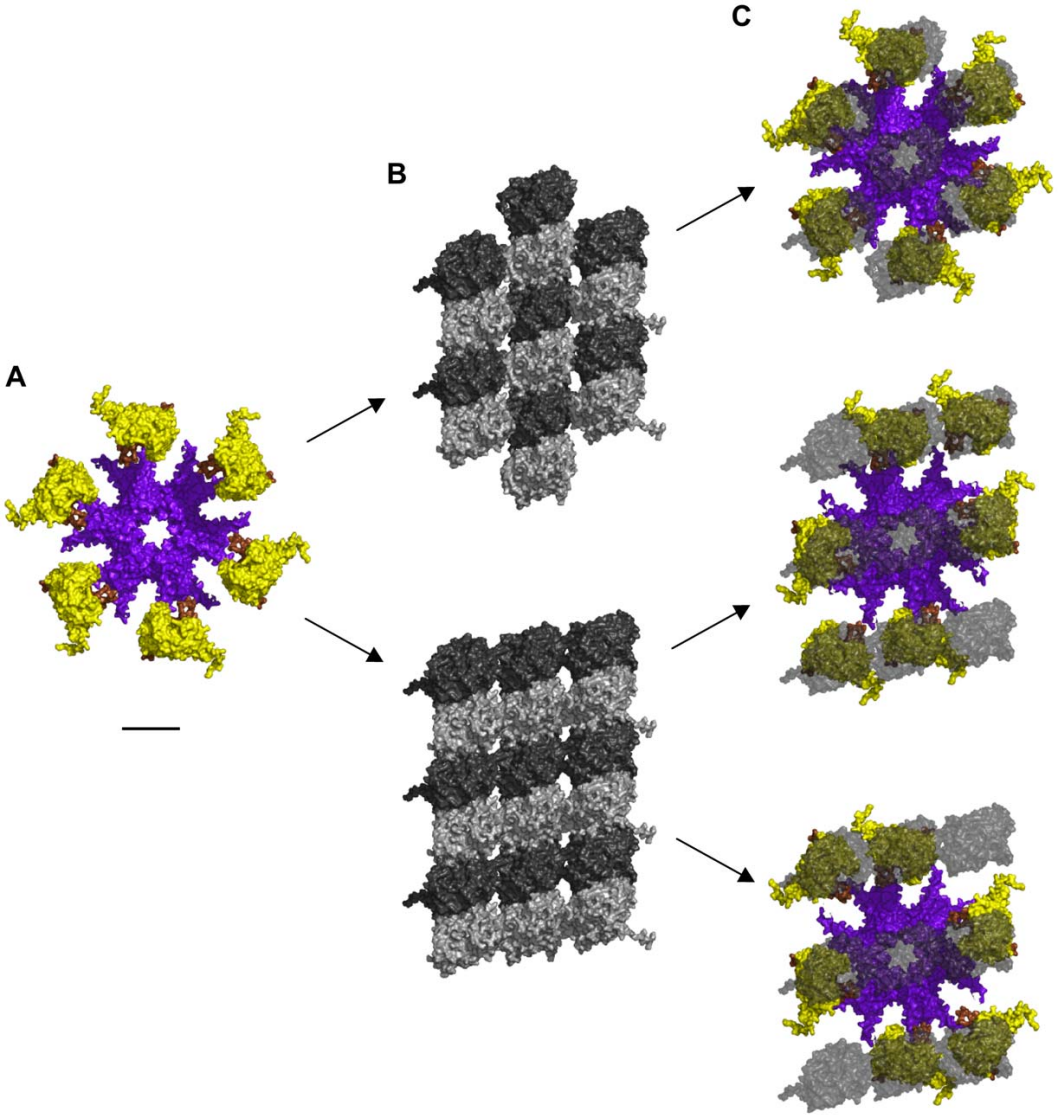
Alignment of CaMKII holoenzyme with MT lattices. (A) Unaligned CaMKII holoenzyme without MT shown from below. Upper kinases and legs not shown. (B) Top – A-lattice patch. Bottom – B-lattice patch, view from the MT lumen looking out. (C) Aligned CaMKII holoenzyme with transparent overlay of tubulin. α-tubulin not shown. Upper – CaMKII on A-lattice MT, Middle – CaMKII on B-lattice MT in configuration 1, Bottom - CaMKII on B-lattice MT in configuration 2. Scale bar 10 nm.

**Figure 4:**
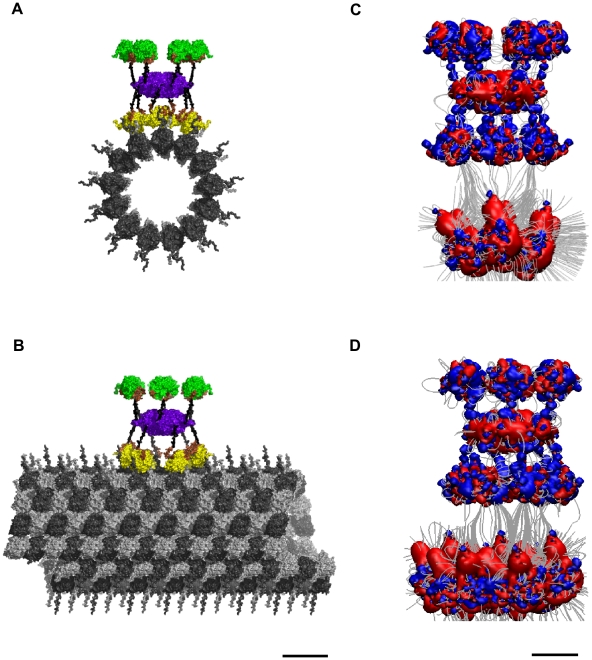
Potential binding of CaMKII to an A-lattice MT. (A) Face view, and (B) Side view of molecular surface. (C) Face view, and (D) Side View of electrostatic map: Blue +0.5 kT/e, Red −0.5 kT/e, Gray – Electric field lines. Scale bar 20 nm.

Size and geometry of the activated hexagonal CaMKII holoenzyme and the two types of hexagonal lattices (A and B) in MTs are identical (see Figure 3). The CaMKII hexagon is approximately 20 nm in breadth (see Figure 3 A), and the maximum distances between individual tubulin monomer in both the A lattice and B lattice MT neighborhoods are the same (see Figure 3 B). With minimal realignment in the linkers between association and kinase domains to account for MT curvature and lattice asymmetry, CaMKII precisely matches the MT A-lattice geometry, i.e. 6 extended kinases can interface collectively with 6 tubulins (see Figure 3 C – upper panel). Overlaying CaMKII with the 9-tubulin neighborhood of the MT B lattice requires slightly greater flexibility to precisely match the geometry (see Figure 3 C – middle and lower panels). This geometric matching permits activated CaMKII holoenzymes to bind to MT surfaces (see Figure 4 A & B).

### Electrostatic Complementarity of CaMKII and MT Lattices

The electrostatic pattern formed by a neighborhood of tubulin dimers on a MT surface (see Figure 2 C & D) shows highly negative charged regions surrounded by a less pronounced positive background, dependent on the MT lattice type (see Figure 2 C & D). These electrostatic fingerprints are complementary to those formed by the 6 CaMKII holoenzyme kinase domains making the two natural substrates for interaction. Alignment of the CaMKII holoenzyme with tubulin dimers in the A-lattice MT arrangement yields converging electric field lines indicating a mutually attractive interaction (see Figure 4 C & D). Considering the positive potential region of an individual kinase domain to be 1 kT/e and the negative surface of an individual tubulin monomer to be −10 e gives an electrostatic attraction of 10 kT, or 6 kcal/mol at 310 K, for each kinase-monomer interaction, the lower bound of binding. This attraction reaches a maximum bound of 60 kT (36 kcal/mol) when all 6 kinase regions are involved. This range of attraction is much stronger than thermal vibrational energy and indicates a significant association.

### Information Storage Capacity

If each extended kinase can either phosphorylate at the S-T site on a tubulin substrate, or not, the process effectively conveys one bit of information (e.g. no phosphorylation = 0, phosphorylation = 1). Each set of six extended kinases on either side of a CaMKII holoenzyme can thus act collectively as 6 bits of information. Ordered arrays of bits are termed ‘bytes’.

**Figure 5:**
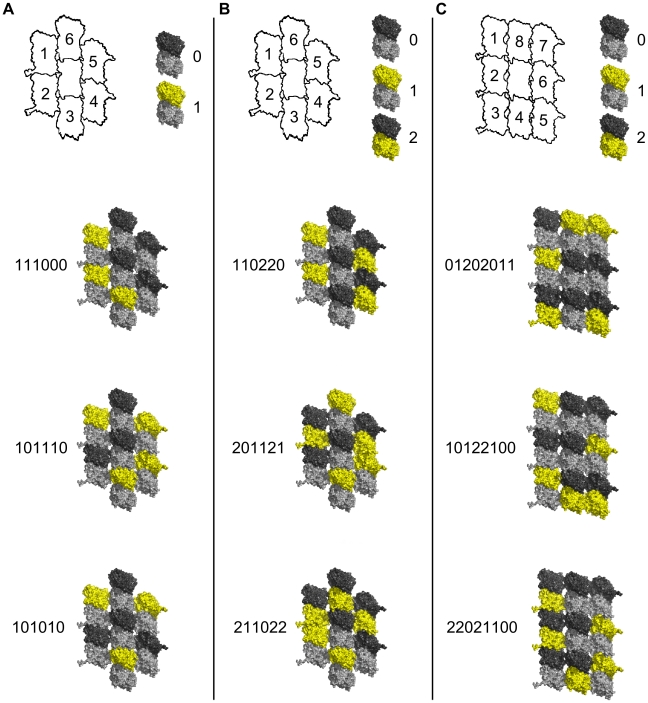
Information capacity in MT lattice due to CaMKII phosphorylation. Top row - schematic tubulin patches with individual dimers identified by numbers (central dimer neglected), and phosphorylation/information states (yellow) of individual dimers with binary or ternary representations. Below - three sample patches with possible phosphorylation states in red. (A) A-lattice patches with β-tubulin binary states. (B) A-lattice patches with ternary states. (C) B-lattice patches with ternary states.

Three hypothetical scenarios for CaMKII information encoding in MT lattice are considered (see Figure 5). In the first situation only β-tubulins in the 7-tubulin neighborhood patch of an A-lattice MT may be phosphorylated, giving two possible states – no phosphorylation (0), and phosphorylation (1) (see Figure 5 A). The scenario would be identical if only α-tubulin phosphorylation was considered. The central dimer is not considered for phosphorylation, so 6 dimers are available. Thus, there are 2^6^ possible encoding states for a single CaMKII-MT interaction resulting in the storage of 64 bits of information. This case, however, only accounts for either α- or β-tubulin phosphorylation, not both. In the second scenario each tubulin dimer is considered to have three possible states – no phosphorylation (0), β-tubulin phosphorylation (1), or α-tubulin phosphorylation (2) (see Figure 5 B). These are ternary states, or ‘trits’ (rather than bits). Six possible sites on the A-lattice yield 3^6^ = 729 possible states. The third scenario considers the 9-tubulin B-lattice neighborhood with ternary states. As in the previous scenarios the central dimer is not considered available for phosphorylation. In this case, 6 tubulin dimers out of 8 may be phosphorylated in three possible ways. The total number of possible states for the B lattice neighborhood is thus 3^6^–2^8^−8(2^7^) = 5281 unique states.

### Bioenergetics

Each phosphorylation event requires hydrolysis of a single ATP molecule, releasing ∼20 kT, or 12 kcal/mol of free energy at physiological temperature for a single kinase-monomer interaction, suggesting a robust molecular mechanism for information encoding. The daily energy usage of the human body requires the production of more than 2×10^26^ molecules of ATP [37]. Brain processes consume approximately one fifth this amount using 5×10^20^ ATP molecules per second. During a calcium influx there are ∼100 CaMKII subunits activated/phosphorylated per synaptic dendrite [38], each using a single ATP molecule. Taking calcium influx signals at a frequency of 100 Hz, activating 100 CaMKII subunits per synapse, and ∼10^4^ synapses per neuron, one neuron would require 10^8^ ATP/second for maximal encoding. With 10^11^ neurons in the brain this amounts to 10^19^ ATP/second, equivalent to 10^19^ bits, at a cost of approximately 2% of the brain's total energy consumption.

Metabolic energy for the proposed memory encoding by CaMKII phosphorylation of MTs should be distinguished from significantly higher metabolic costs of more coarse-grained forms of neuronal information processing. For example, hydrolysis of 10^4^ ATP molecules is required to transmit one bit of information at a chemical synapse, and hydrolysis of 10^6^ to 10^7^ ATPs is needed for graded signals or spike coding [39].

## Discussion

We presume memory encoded by CaMKII phosphorylation alters, programs and provides a background for ongoing MT-based information processing e.g. microtubule automata, finer-grained processes underlying membrane potentials and synaptic activities. Information processing in post-synaptic dendritic and somatic MTs can thus: 1) integrate inputs to firing threshold at the proximal axon, and 2) regulate synaptic plasticity. In the following, we describe 6 possible mechanisms by which CaMKII-encoded tubulin phosphorylation in MT lattices could regulate cytoplasmic, membrane and neuronal structure and function leading to cognition and behavior.

### C-Terminal Tail Extension

**Figure 6:**
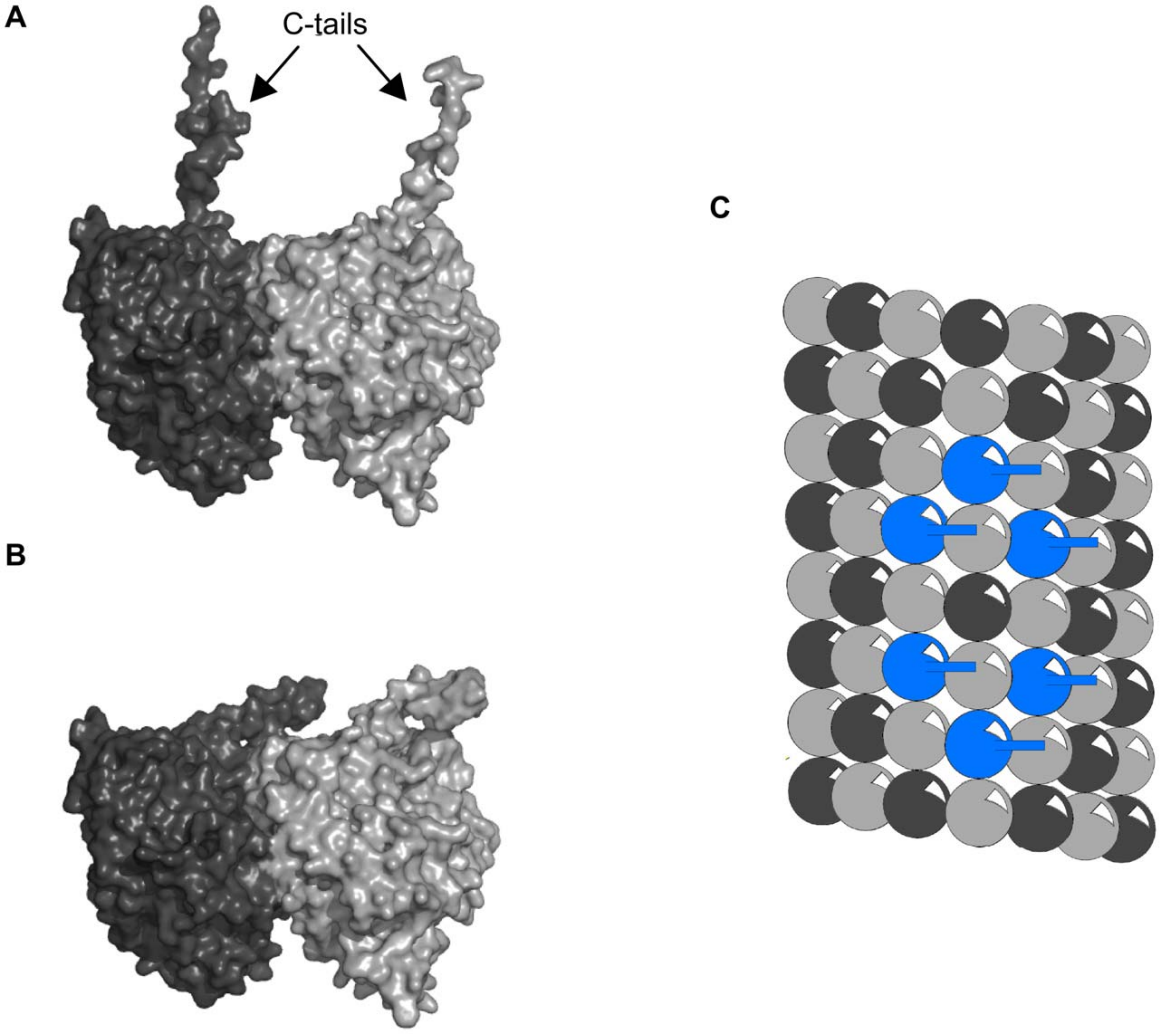
Tubulin C-terminal tail states. (A) Up-state. C-terminal tails of α- and β-tubulin extended into the cytosol. (B) Down-state. C-terminal tails folded and interacting with the tubulin body. (C) Light grey sphere - α-tubulin tail down, Dark grey sphere - β-tubulin tail down, Blue sphere – β-tubulin tail up.

Each tubulin dimer has two C-terminal ‘tails’, one from each monomer (see Figure 6). Modeling has shown that C-terminal tails can exist in multiple states, and that the tails may dynamically oscillate between extended conformations, involving interaction with water and ions (the “up-state”) (see Figure 6A), and folded conformations, involving interactions with the tubulin body (the “down-state”) (see Figure 6B) [40]. Thus, under proper conditions the negatively charged tails extend outward into the cytoplasm, able to alter local electric fields, as well as ionic and chemical states. CaMKII-induced phosphorylation of particular tubulins may cause dynamical (e.g. hexagonal) patterns of C-terminal extension, able to precipitate reaction-diffusion waves. Reaction-diffusion patterns known as Turing patterns [41], [42] are thought to function in intra-cellular information processing.

### MAP Attachment Sites

**Figure 7:**
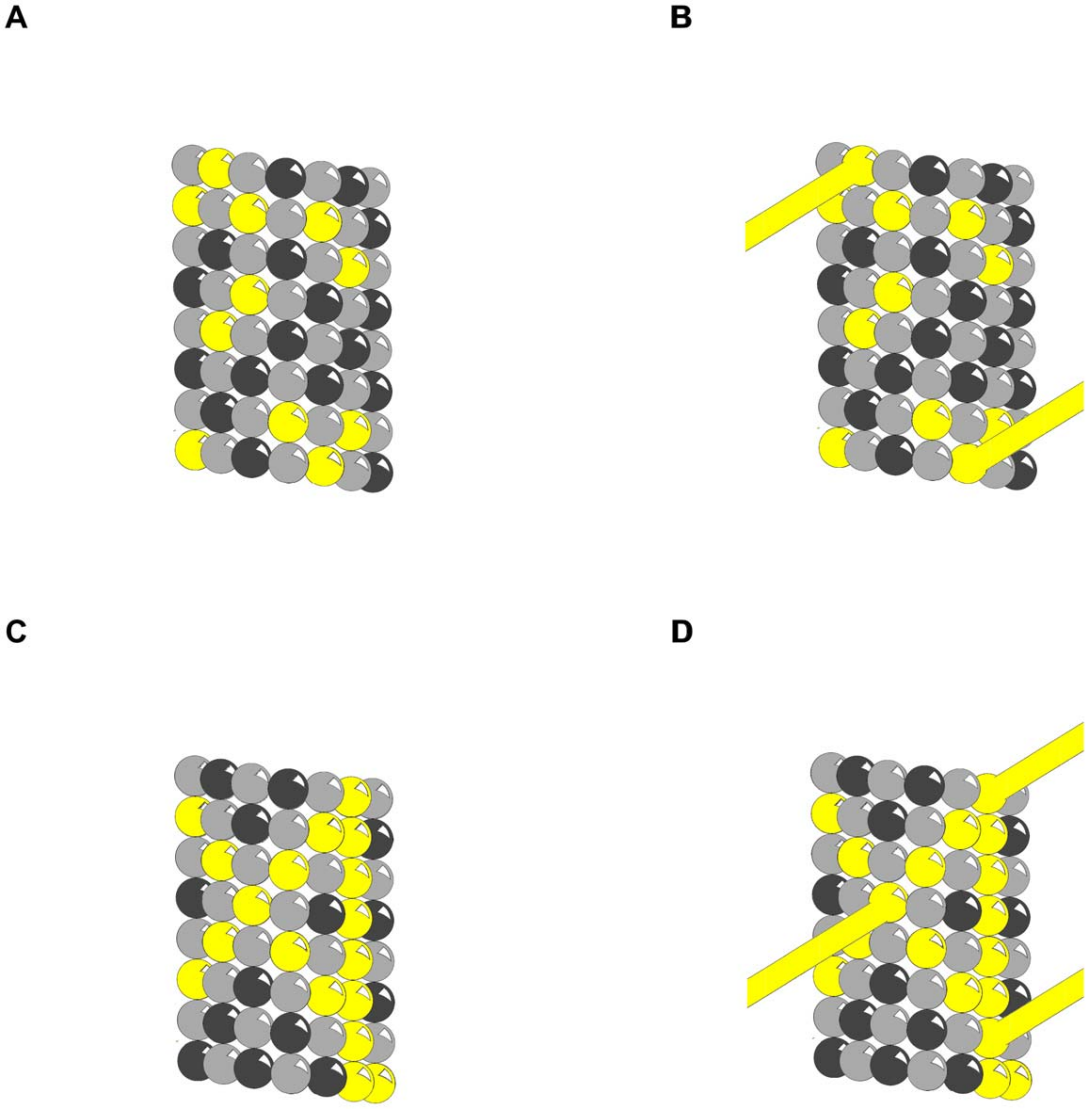
Phosphorylation patterns determine MAP attachment. Light grey sphere - α- tubulin, Dark grey sphere - β-tubulin, Yellow sphere – phosphorylated tubulin, Yellow cylinder – MAP. (A) Phosphorylation pattern on microtubule. (B) Hypothetical MAP attachment sites (phosphorylated tubulin in (A) neighbored by 2 phosphorylated tubulin indicates MAP attachment). (C) Phosphorylation patterns follow MT lattice geometry (protofilament, helical, anti-helical. (D) Intersection points in (C) indicate MAP attachment sites.

MAPs bind on the surface of individual tubulins in the region where phosphorylation occurs [27], [39], and removal of phosphate groups on tubulin results in decreased MT assembly in the presence of MAP2 [43]. These findings suggest that tubulin phosphorylation promotes MAP-MT interaction [27]. CaMKII induced phosphorylation patterns on MTs would thus both promote MAP-MT binding in general, and also serve as templates for attachment of MAPs at specific sites. MAPs attached in this way can form bridges between MTs, and thus cytoskeletal scaffolding determining MT spacing, neuronal architecture and synaptic location (Figure 7 A & B).

Figure 7 C shows pathways of phosphorylated tubulins following MT lattice geometry, intersecting at various tubulin sites. In Figure 7 D the intersection points are sites for MAP attachment. Patterns of phosphorylated tubulins can determine cytoskeletal morphology and cellular function.

In the absence of CaMKII, MAPs bind to MTs *in vitro* in super-helical lattice geometric patterns [44]–[46]. These may reflect resonance nodes of MT lattice vibrations with which CaMKII phosphorylation and other information modes interact.

### Motor Protein Transport

**Figure 8:**
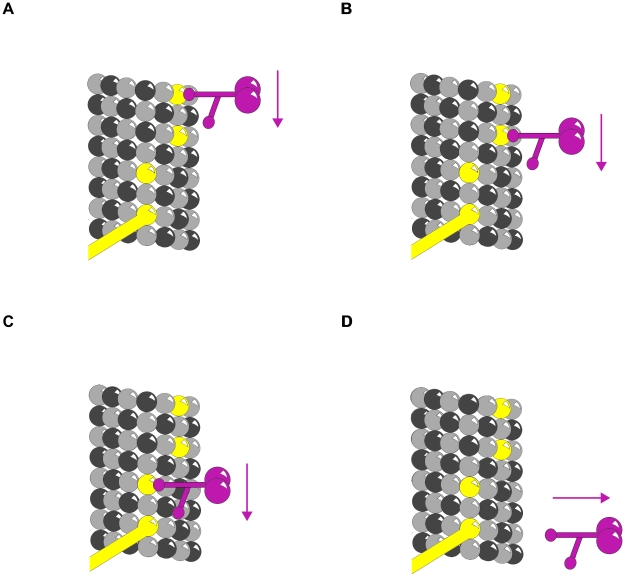
Phosphorylation patterns direct motor proteins. Purple – Motor protein. (A)–(C) Three time frames showing motor protein following phosphorylated tubulin pathway. (D) MAP directs motor protein to disengage microtubule and deliver cargo.

Motor proteins dynein and kinesin move (in opposite directions) along MTs (using ATP as fuel) to transport and deliver components and precursors to specific synaptic locations. While MTs are assumed to function as passive guides, like railroad tracks, for motor proteins, the guidance mechanism by which they follow specific paths, e.g. through branching dendrites and interrupted MTs is unknown. Phosphorylation patterns on MT lattices could guide motor proteins to specific locations (see Figure 8 A–C).

Motor protein transport along MTs also depends on other types of MAPs, e.g. optimally spaced between ∼15 and 25 nm (equivalent to the length of two to three dimers) apart along the protofilament length [47], [48]. MAP tau (whose hyperphosphorylation and detachment from MTs correlates with Alzheimer's disease) appears to signal motor proteins precisely where and when to disengage from MTs and deliver their cargo [49] (see Figure 8 D). Consequently, patterns of CaMKII-induced tubulin phosphorylation in MT lattices could regulate motor protein transport along MTs directly, and/or by MAP attachment locations.

### Signaling and Computation

Cytoskeletal structures including MTs were first suggested to process information and regulate cellular function by Sir Charles Sherrington [16]. Computational interaction among discrete tubulin states [18], [50], and signals propagating along MT protofilaments and/or helical winding pathways have been proposed [15] (e.g. in dendritic and somatic integration in ‘integrate-and-fire’ brain neurons, regulating axonal firings and synaptic plasticity [51]).

Both MTs and MAPs can support solitary waves of ionic transport flow, e.g. via C-terminal tails on MT exteriors [52]. Signals may also propagate through MTs by intra-tubulin conduction pathways defined by electron resonance rings of aromatic amino acids [15]. Evidence suggests highly conductive electron and/or soliton conductance through MT lattices, including via helical pathways [53]. Signaling has also been proposed to occur in MT hollow cores [54].

MTs appear to have intrinsic electro-mechanical vibrations, e.g. coherent oscillations in the low megahertz range [55]. MT assembly is enhanced by 6 orders of magnitude when exposed to an applied 1 to 3 megahertz (radio frequency) alternating current, emits laser-like 3.1 to 3.8 megahertz radiation, and exhibits some form of electron condensation [53]. Signal propagation will be influenced by, and depend on MT lattice vibrations and related effects.

### Frequency Dependence of Inputs

Dynamic interplay among CaMKII phosphorylation, communicative signaling/vibration, and MAP attachment sites are rich opportunities for regulation of synapses and intra-neuronal processes. Analogies to musical instruments have been suggested, e.g. MAP attachments acting as ‘frets’, as in a guitar [56], or the “guitar string hypothesis” [57] altering wavelengths and changing MT resonant frequency with consequences for functional processes.

In LTP, high frequency inputs (e.g. 50 to 100 Hz) are required for prolonged post-synaptic response. Kumar et al [58] showed that memory formation in dendrites depends on synchronized inputs, with an optimal frequency near 50 Hz. Density and patterns of CaMKII-induced tubulin phosphorylation in post-synaptic MT lattices would depend on frequency and synchrony of inputs.

### Logic Gates

**Figure 9:**
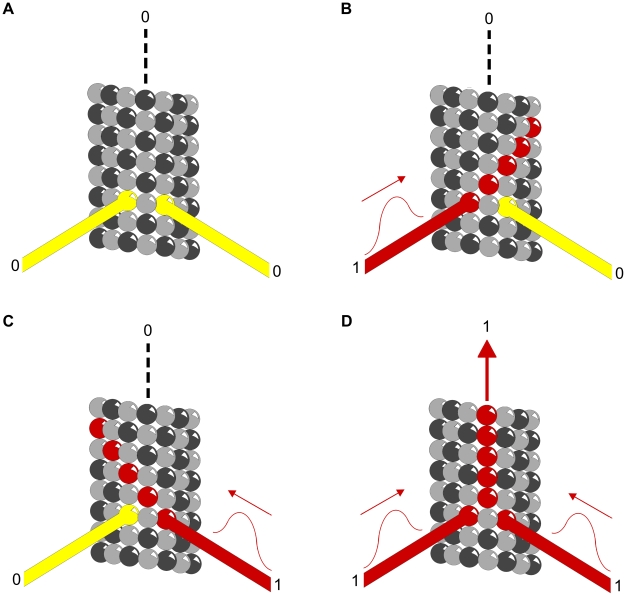
Truth table for AND gate with MAPs as input. Red – propagating signal. MAPs are input and central protofilament is output. (A) (0,0→0) No input along either MAP yields no output along the protofilament. (B) (1,0→0) Input along one MAP continues along helical path with no output along protofilament. (C) (0,1→0) Input along one MAP continues along anti-helical path with no output along protofilament. (D) (1,1→1) Input along both MAPs arrive simultaneously canceling lateral contributions sending a signal along the protofilament.

**Figure 10:**
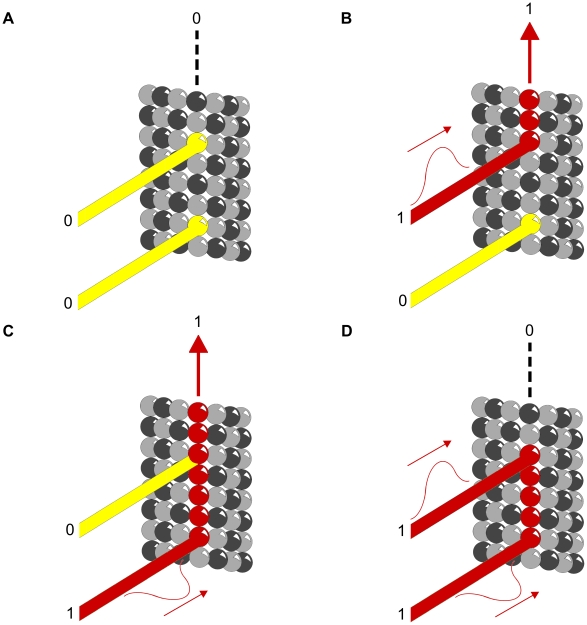
Truth table for XOR gate with MAPs as input. Red – propagating signal. MAPs are input and central protofilament is output. (A) (0,0→0) No input along either MAP yields no output along the protofilament. (B) (1,0→1) Input along one MAP sends signal along protofilament. (C) (0,1→1) Input along one MAP continues sends signal along protofilament. (D) (1,1→0) Input along both MAPs out of phase cancel sending no signal along the protofilament.

**Figure 11:**
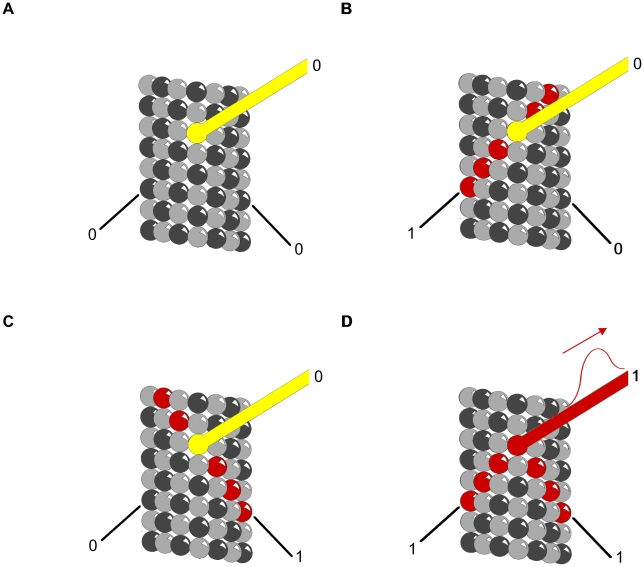
Truth table for AND gate with MAPs as output. Red – propagating signal. Helical and anti-helical tubulin paths are input and MAP is output. (A) (0,0→0) No input along either helix yields no output along the MAP. (B) (1,0→0) Input along helix continues along helix with no MAP output. (C) (0,1→0) Input along anti-helix continues along anti-helix with no MAP output. (D) (1,1→1) Input along both helices arrive simultaneously in phase canceling lateral contributions sending a signal along the MAP.

**Figure 12:**
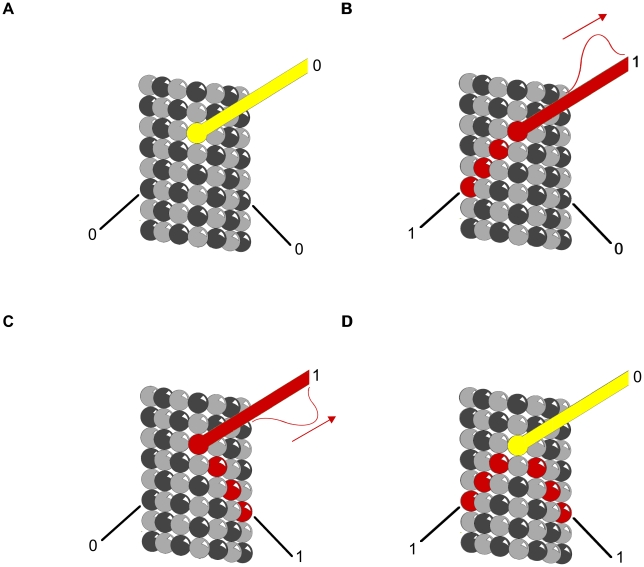
Truth table for XOR gate with MAPs as output. Red – propagating signal. Helical and anti-helical tubulin paths are input and MAP is output. (A) (0,0→0) No input along either helix yields no output along the MAP. (B) (1,0→1) Input along helix continues along MAP as output signal. (C) (0,1→1) Input along anti-helix continues along MAP as output signal. (D) (1,1→0) Input along both helices arrive simultaneously out of phase canceling the signal along the MAP.

Clusters of phosphorylated tubulin, and/or MAP attachment may serve as logic gates for propagating information. Figures 9 and 10 demonstrate two types of Boolean logic gates, an AND gate and an exclusive OR gate (XOR) in which MAPs convey inputs, with output along tubulin pathways. Figures 11 and 12 show AND and XOR gates in which MAPs convey output of inputs and processes in tubulins within the MT. The combination of XOR and AND logic gates forms a universal set for computation in which all other logic gates (NOT, OR etc.) can be conceived. Signals propagating through MT-MAP logic circuits may extend throughout cytoskeletal networks, regulating synaptic function, cognition and behavior.

### Summary

‘Encoding’ is conversion of information from one form to another, each form requiring a geometry in which information is represented, e.g. as ‘bits’. Computer pioneer Alan Turing described a one-dimensional geometry, a string of information on a linear tape. Another computer pioneer John Von Neumann and others [59] developed two-dimensional cellular automata, lattice surfaces of interactive ‘cells’, or units. Simple rule-based interactions among neighbor cell states updating at discrete time steps can lead to complex patterns, computation and self-organization in cellular automata. Recently, three-dimensional assemblies of duroquinone molecules have been shown to function as ‘molecular’ automata (‘nanobrains’) [60].

Biology uses dynamical three-dimensional information via membrane dynamics, concentration gradients of various cytoplasmic components (e.g. reaction-diffusion) and cytoskeletal processes. How information is shared or encoded among these levels in a common framework is largely unknown.

We address information processing in cytoskeletal MTs, polymers of the protein ‘tubulin’, in both A-lattice and B-lattice configurations. Under proper conditions tubulin self-assembles into MTs, cylindrical hexagonal lattices, which directly participate in cell organization. Sherrington in 1951 [16] first proposed the cytoskeleton might serve as a cellular ‘nervous system’, and others have suggested microtubule-based information processing, e.g. with individual tubulins representing bit-like information states [18], [19], [56]. For example MTs have been modeled as von Neumann-type cellular/molecular automata (‘microtubule automata’) in which tubulin subunits interact with neighbor tubulin states by rules based on dipole coupling strengths [50], [61]. In such proposed microtubule automata, tubulin states interact and update coherently at discrete time steps attributed to theorized [62] or experimentally-observed coherent MT resonances, e.g. in the low megahertz range [53], potentially resulting in millions of synchronized updates per second [63].

To simulate microtubule automata, two-dimensional hexagonal MT lattices are slightly skewed according to lattice geometry and wrapped into a three-dimensional cylinder. Simulations show oscillating wedge-shaped, triangular and hexagonal patterns of tubulin states, which evolve, compute and can learn [50]. Because of cylindrical MT lattice geometry, such patterns reverberate, interfere and change nonlinearly. Thus microtubule automata are in principle capable of information processing. How could they interact with membranes and beyond, with extra-cellular processes? How could microtubule automata receive inputs and express outputs?

In this paper we evaluated possible information inputs to microtubules in the context of brain neuronal memory encoding and long-term potentiation (LTP). A key intermediary in LTP involves the hexagonal holoenzyme calcium-calmodulin kinase II. When activated by synaptic calcium influx, the snowflake-shaped CaMKII extends sets of 6 foot-like kinase domains outward, each domain able to phosphorylate a substrate or not (thus convey 1 bit of information). As CaMKII activation represents synaptic information, subsequent phosphorylation by CaMKII of a particular substrate may encode memory, e.g. as ordered arrays of 6 bits (one ‘byte’). We used molecular modeling to examine feasibility of collective phosphorylation (and thus memory encoding) by CaMKII kinase domains of tubulins in a microtubule lattice.

We show, first, complementary electrostatics and mutual attraction between individual CaMKII kinase domains and tubulin surfaces. We also demonstrate two plausible sites for direct phosphorylation of tubulin by a CaMKII kinase domain, and calculate binding energies in the range of 6 to 36 kcal/mol per CaMKII-tubulin phosphorylation event. This indicates encoding which is robust against degradation, yet inexpensive, requiring on the order of 2% of overall brain metabolism for maximal encoding in all 10^11^ neurons.

We then compare size and hexagonal configuration of the six extended foot-like kinase domains of activated CaMKII with hexagonal lattices of tubulin proteins in MTs. We find that CaMKII size and geometry of 6 extended kinase domains precisely match hexagonal arrays of tubulin in both A-lattice and B-lattices.

We quantified the potential information capacity for CaMKII hexagonal encoding of MT lattice regions, specifically in lattice ‘patches’ of 7 (A lattice) or 9 (B lattice) tubulin protein dimers. The simplest case was taken as CaMKII phosphorylation of an A lattice patch of 7 tubulin dimers (central ‘address’ dimer unavailable for phosphorylation, 6 available dimers). Each kinase domain can phosphorylate one tubulin dimer, either its α- monomer or its β- monomer equivalently. For either dimer, phosphorylation = 1, no phosphorylation = 0. Sets of 6 CaMKII kinase domains interacting with 6 tubulin dimers can then provide 6 binary bits (64 possible states), comprising one byte.

On an A lattice we also consider each tubulin dimer being phosphorylated either on its α-tubulin monomer (0), α-tubulin monomer (1), or neither (2), resulting in 6 ternary states (‘trits’) or 729 possible encoding states (per ‘tryte’).

We also considered ternary states in a B lattice with 9 tubulin dimers in a patch. With the central ‘address’ dimer unavailable for phosphorylation, sets of 6 CaMKII kinase domains can choose and phosphorylate α- (0), β- (1), or neither (2) tubulin monomer in any 6 of 8 available dimers. This yields 5,281 possible information states per neighborhood patch of 9 tubulin dimers. There are approximately 10^19^ tubulins in the brain. Consequently, potential information capacity for CaMKII encoding of hexagonal MT lattices is enormous.

Assuming input information may be encoded and processed in hexagonal microtubule lattices, how could such information be expressed as output to regulate cytoplasmic and membrane activities? We list six possible mechanisms including a potential connection to reaction-diffusion systems in cytoplasm.

Concentration gradients of various molecules in cytoplasm regulate cellular activities. In 1953 Alan Turing [64] showed that stable patterns of molecular concentrations could emerge through reaction, diffusion and inhibition. Reaction-diffusion systems include periodic waves and hexagonal patterns, which oscillate, undergo phase transitions and are capable of information processing [41]. Turing patterns have been suggested in biological systems across scale, e.g. from intra-cellular processes [42] to neuronal network organization. Cortical neurons representation maps are organized hexagonally [65], and ‘grid cells’ in entorhinal cortex record spatial location as apparent hexagonal maps, with different spatial scales at different layers of entorhinal cortex [66], [67].

‘Scale-free’ implies self-similar information patterns repeating at different spatial and temporal scales (following 1/frequency power laws). Similar to ‘fractals’ and holograms, scale-free structures and processes arise commonly in nature and technology, and are inherently robust and resistant to disruption. In the brain, evidence suggests neuronal network structure, temporal dynamics, and representation of mental states are all ‘scale-free’, with self-similar patterns repeating at various temporal and spatial scales and locations [68]–[71]. Interference patterns of periodic, coherent reaction-diffusion waves in cytoplasm and larger spatial scales could account for scale-free information patterns regulating biological systems including the brain. Microtubules can generate three-dimensional reaction-diffusion patterns [70], and we suggest such patterns operate at multiple time scales to regulate biological systems.

### Conclusion

We demonstrate a feasible and robust mechanism for encoding synaptic information into structural and energetic changes of microtubule (MT) lattices by calcium-activated CaMKII phosphorylation. We suggest such encoded information engages in ongoing MT information processes supporting cognition and behavior, possibly by generating scale-free interference patterns via reaction-diffusion or other mechanisms. As MTs and CaMKII are widely distributed in eukaryotic cells, the hexagonal bytes and trytes suggested here may reflect a real-time biomolecular information code akin to the genetic code.

## Methods

### Calcium Calmodulin Kinase II Holoenzyme Modeling

Sequences of human αCaMKII kinase, autoregulatory, linker, and association domains, as defined by Dosemici et al. [72] and Tombes et al. [73] were used to build homology models. Crystal structures 1HKX [74] and 2VZ6, found in the Protein Data Bank (PDB) [75], were used as templates to build basic homology models of the association, kinase and autoregulatory domains, respectively, using MODELLER 9V6 [76]. The CaMKII holoenzyme structure was built with PYMOL 0.99rc6 [77] using the geometry described in Rosenberg et al. [78] with the linker region constructed as a linear chain of residues joining the autoregulatory and association domains.

### Tubulin and Microtubule Lattice Modeling

PDB tubulin protein structure 1JFF [79] was repaired by adding missing residues from 1TUB [80]. The repaired 1JFF dimer was solvated, neutralized and energy-minimized using NAMD [81]. The minimized and repaired 1JFF structure was used as a template to build basic homolgy models of TUBA1A and TUBB3 using MODELLER 9V6 [82]. Using this dimer, MT A and B lattice structures were built with PYMOL 0.99rc6 [77] using MT geometry described in Li et al. [82] and Sept et al. [32]. PYMOL 0.99rc6 [77] was used to model and illustrate the positional geometry changes of CaMKII-tubulin/MT lattice interactions.

### Electrostatic Maps and Field Lines Modeling

To analyze electrostatic matching, hydrogens were added, and protonation states set at pH 7 with PROPKA [83], via PDB2PQR [84], [85] for both CaMKII and MT lattice structures. The Poisson-Boltzmann equation was solved for the structures in given arrangement with the Adaptive Poisson-Boltzmann Solver (APBS) [86] at a grid spacing of less than 1 Å. Isosurfaces were generated at +0.5 kT/e and −0.5 kT/e, and field lines were drawn with gradient magnitude 3. Electrostatic and field line images were generated in PYMOL 0.99rc6 [77] and VMD 1.8.7 [87], respectively.
